# Socioeconomic status and motor coordination function among children with autism

**DOI:** 10.3389/fpsyt.2025.1619918

**Published:** 2025-11-26

**Authors:** Muqing Cao, Tingfeng Gu, Xiuhong Li, Jin Jing

**Affiliations:** 1 Department of Sport Medicine, Guangzhou Sport University, Guangzhou, China; 2 Guangzhou Center for Disease Control and Prevention, Guangzhou, China; 3 Sun Yat-sen University (Shenzhen), Shenzhen, China; 4 Sun Yat-sen University, Guangzhou, China

**Keywords:** autism, motor impairment, socioeconomic factors, motor coordination disorder, maternal education, family income

## Abstract

**Objective:**

To examine the association of socioeconomic status with the motor coordination impairment (probable DCD) of autism in a Chinese population.

**Methods:**

Using cross-sectional data from the south region of China, 2020-2021, per capita family income, maternal education, and whether the only child was included as the indicators of socioeconomic status, motor coordination function was evaluated by the Developmental Coordination Disorder Questionnaire (DCDQ). We included 165 autistic children aged 2–12 years in the final analysis, among whom 88 were classified as probable DCD.

**Results:**

After adjusting for covariates, the odds ratios for probable DCD among autistic children whose per capital family income was below 5000 yuan (OR: 4.77, 95% CI: 1.71-13.30) or 5000–8000 yuan (OR: 3.62, 95% CI: 1.36-9.61) per month were significantly higher than children whose per capital family income above 8000 yuan per month. Mothers without a college degree showed a decreased likelihood of probable DCD (OR: 0.33, 95%CI: 0.15-0.71).

**Conclusion:**

Low income of a family is associated with a higher risk of probable DCD among autism children. The linkage between higher education of the mother and probable DCD could be attributed to more grandparenting among these families. This is the first research exploring the association between socio-demographic factors and the motor coordination function of autism. It comes from a set of low/middle-income countries with limited sources of autism in the Asian area, and comprehensive SES factors are considered. We recommend more support to autism children from low-income families, especially motor-related intervention.

## Introduction

In addition to core symptoms encompassing social communication challenges and restricted/repetitive behaviors, emerging evidence highlights concurrent motor dysfunction in children with autism spectrum disorder (ASD). Epidemiological studies from the US report early motor delays in 50-89.7% of children with ASD (1) and persistent motor coordination deficits (2) within this population (3). These robust findings have prompted scholarly proposals to recognize motor impairment as a distinct diagnostic criterion for ASD (4), underscoring the critical need for intensified research into motor development trajectories in ASD.

While evidence of motor dysfunction in ASD is well-established, research examining its determinants, particularly the role of socioeconomic status (SES), remains limited with little theoretical framework (5). Preliminary evidence suggests bidirectional associations between motor impairments and both core ASD symptoms (social communication deficits, restricted/repetitive behaviors) and comorbid intellectual disability (4, 6, 7). Notably, early motor dysfunction demonstrates predictive validity for subsequent ASD diagnosis (2), implying potential neurodevelopmental substrates shared across motor and socio-behavioral domains (7). While the etiology of motor coordination deficits in ASD remains underexplored, emerging data implicate socioeconomic status (SES) as a multifactorial determinant encompassing family resources (8, 9), environmental enrichment, and access to health interventions (10)., as systematically reviewed in **eTable 1**. However, the potential pathways linking specific socioeconomic status (SES) factors to motor development in autism spectrum disorder (ASD) remain poorly understood. This gap, coupled with the lack of a cohesive theoretical explanation, challenges the formulation of future mechanistic hypotheses.

Bronfenbrenner’s bioecological model, with its emphasis on multi-level, process-oriented interactions, serves primarily as ​an interpretive lens and a heuristic framework​ for this study (11). While the complex, nested structures of the ecosystem (from micro- to macro-system) provide a comprehensive conceptual map for contextualizing the potential pathways through which SES may influence motor development, our empirical measurement focuses on key distal SES indicators (household income and maternal education). This approach allows us to ​establish foundational associations​ within a clinically accessible sample. The rich theoretical framework is thus employed as a ​critical interpretation of our findings within a broader ecological context and to generate nuanced hypotheses for future research​ that can directly measure process-level mechanisms across all ecological levels (11).

The relationship between SES and motor development in neurotypical populations is also complex and multifaceted, which can be further illuminated through an ecological lens.​​ Meta-analytic evidence consistently links higher SES to enhanced motor proficiency across developmental domains: motor competence (12–14), gross motor skills (15–17), fine motor precision (9, 18, 19), motor coordination (13, 20), and processing speed (21). However, the finding that ​elevated maternal education was associated with motor delays​ (22) presents a notable contradiction to the prevailing literature and suggests that in certain contexts, ​this relationship can be inverted. This inversion ​challenges the assumption of a universally positive linear association​ and underscores the possibility that ​the mechanisms linking SES to motor development are more complex and context-dependent than previously appreciated. On the other hand, null associations between SES and motor outcomes (21, 23–25).

This inconsistency underscores the need for population-specific investigations, particularly given the critical knowledge gap regarding SES-motor relationships in children with neurodevelopmental conditions like ASD.

Building on this bioecological framework, we investigated the associations between SES and motor function in a large, clinic-based sample of Chinese children with ASD aged 2–12 years.​​ Our study aims to provide the first empirical evidence of such social inequalities in this population by: (a) quantifying the relationships between key socioeconomic markers (household income per capita, maternal education) and motor dysfunction prevalence; and (b) examining whether these associations persist after controlling for core developmental covariates, including cognitive capacity, handedness, and ASD symptom severity. Furthermore, sex-based comparisons are critical in ASD research due to the well-documented ​sex disparity in prevalence rates, with males being diagnosed significantly more frequently than females (26). Beyond prevalence, emerging evidence suggests potential ​sex differences in phenotypic expression, including motor skills. Therefore, examining socioeconomic factors in relation to motor dysfunction separately by sex can help elucidate whether risk factors operate similarly or differently across groups.

We hypothesized that lower SES would be associated with a higher risk of motor impairment, even after accounting for these potential confounders.​​ To our knowledge, this is the first study to examine SES-motor relationships in ASD through a bioecological lens while simultaneously adjusting for key neurodevelopmental factors. Our findings aim to inform the development of targeted early interventions and public health policies designed to mitigate developmental disparities in ASD populations.

## Methods

### Study population, overall design, and procedure

This cross-sectional investigation was conducted across three rehabilitation centers in Southern China from October 2020 to August 2021. Participants comprised 168 children with autism spectrum disorder (ASD) aged 2–11 years (M = 5.3 ± 2.1), meeting the following inclusion criteria: (1) confirmed ASD diagnosis per DSM-5 criteria through multidisciplinary clinical evaluation; (2) absence of severe sensory impairments (uncorrected visual acuity >20/200, hearing loss >40 dB HL) or neuromotor disabilities affecting assessment validity. The institutional review board at the corresponding author’s affiliated university granted ethical approval (Protocol No. 2019-056). Legal guardians provided written informed consent before data collection, which involved two parallel components:

Parent-reported measures:

1. Sociodemographic characteristics (family income, maternal education).
2. Chinese version of the Developmental Coordination Disorder Questionnaire (DCDQ; sensitivity=0.85, specificity=0.91).
3. ASD symptom severity via Childhood Autism Rating Scale (CARS; α=0.83).

Clinician-administered assessments:

1. Cognitive functioning using the Chinese-Wechsler Preschool Scale.
2. Laterality evaluation through performance-based handedness tasks.

All assessments were conducted by trained clinicians blind to SES information. Community engagement was not pursued, given the clinical focus of this facility-based study.

### Sample size determination

The sample size for this cross-sectional study was calculated to ensure sufficient precision in estimating the association between socioeconomic status (SES) and motor dysfunction in children with ASD. Based on the primary outcome of motor proficiency (assessed by the DCDQ-Chinese version, a continuous variable), we used the formula for estimating a population mean in a cross-sectional design:


$$n={\left(Z_{\alpha /2}\cdot \sigma /d\right)}^{2}$$

Where:

Z_α/2_​=1.96 (the critical value for a two-sided test at α = 0.05).

σ=12 (the estimated standard deviation of DCDQ scores, derived from prior studies on motor function in ASD children).

d=2 (the margin of error, representing a clinically meaningful difference in DCDQ scores).

The calculation yielded a minimum sample size of:


n=(21.96×12)2=138.3≈139 participants

To account for potential non-response or missing data (estimated at 15%), the target sample size was increased to:


$$n_{adjusted}=1-0.15139\approx 164\;participants$$

Our study aimed to recruit at least 164 participants to achieve 85% statistical power for detecting associations with an effect size of Cohen’s f² ≥ 0.15. The final sample included 166 children, which meet the calculated requirement and thus strengthens the analysis.

### Social demographic characteristics

Sociodemographic data were systematically captured through standardized questionnaire items. Key independent variables included:

​Maternal educational attainment.

Assessed using a multiple-choice item:

“What is the mother`s education level? A. junior middle school or below, B. high school/vocational high school, C. college, D. Bachelor’s degree or above”.

Responses were categorized into four ordinal levels reflecting the Chinese education system hierarchy.

​ Household income per capita was operationalized through the query:

“What is your per capita family income per month? Per capita family income means the income of all family members that live together divided by the number of people living together. Family income includes salary, bonus, and income from investments. A. 5,000 yuan or below, B. 5,000-8,000 yuan, C. above 8,000 yuan”. This classification was designed to align with ​broad income stratification concepts​ commonly referenced in Chinese socioeconomic and health studies, which often define these ranges as indicative of ​low-income, lower-middle-income, and moderate-to-comfortable income levels​, respectively. To contextualize these tiers within the broader national economy, we referenced China’s ​2019 per capita GDP of 10342.9 US dollars​ (annually), as reported by the National Bureau of Statistics (27). When viewed against this national benchmark, our income categories represent meaningful gradients within the country’s socioeconomic landscape: Tier A falls significantly below the national average economic output, Tier B represents an intermediate range, and Tier C, above the median income.

### Motor assessment

Motor coordination assessment employed the Chinese version of the Developmental Coordination Disorder Questionnaire (DCDQ), a validated 17-item parent-reported measure with age-specific subscales. For children aged 6–10 years: 1) Fine Motor/Handwriting Skills, 2) General Coordination, 3)Control During Movement. For children aged 2–5 years: 1) Fine Motor/Handwriting Skills, 2) General Coordination, 3) Control During Movement, 4) Gross Motor/Planning. Both versions yield a composite score (17-85), where elevated scores denote superior motor proficiency. Following established clinical guidelines, we applied a total score threshold of <49 to classify probable developmental coordination disorder (DCD) versus typically developing status (≥49). This cutoff was applied to both age groups (2–5 years and 6–10 years) in accordance with the scoring guidelines for the Chinese versions of the questionnaire utilized in this study (28), aligns with previous validation studies of the Chinese DCDQ, demonstrating 82% sensitivity and 78% specificity for detecting motor impairment (29).

It is important to note that the identification of motor difficulties (herein referred to as ​probable Developmental Coordination Disorder) in this study is based on scores from the Developmental Coordination Disorder Questionnaire (DCDQ), a screening tool, and does not equate to a formal clinical diagnosis based on DSM-5 criteria.

### Evaluation of the severity of the autistic symptom

Autism symptom severity was quantified using the Childhood Autism Rating Scale (CARS), a clinician-administered observational assessment with established diagnostic validity (Schopler et al., 1980). This 15-item instrument evaluates behavioral domains through direct child-clinician interaction, with each item scored on a 4-point continuum: ​1 point: Age-appropriate functioning, ​2 points: Mild atypicality, ​3 points: Moderate impairment, ​4 points: Severe maladaptive behavior. Standardized scoring protocols with operationalized behavioral anchors accompanied each domain to ensure inter-rater reliability. Two doctoral-level clinicians certified in ASD assessment conducted all evaluations after completing 40-hour CARS training, achieving interclass correlation coefficients (ICC) of 0.89 during pre-study calibration. Total scores (15-60) were analyzed as continuous variables in subsequent regression models.

### Potential cofounders

Covariate selection followed evidence-based epidemiological principles, incorporating:

​Demographic factors: Maternal age at childbirth, child’s chronological age, and age at ASD diagnosis. ​Family structure: Singleton status, grandparental caregiving involvement, ​Neurodevelopmental markers: Handedness (laterality index > +0.50 as right-handed), ​Cognitive functioning: Quantified through standardized assessments. All covariates except cognitive capacity were ascertained via a structured parental interview. Handedness, as a proxy for cerebral lateralization, was included as a covariate because ​atypical patterns of handedness​ are more frequently observed in individuals with ASD and have been linked to differences in motor coordination and neurological organization (30). By controlling for handedness, we aimed to better isolate the specific relationship between SES and motor skills.

Cognitive evaluation employed age-stratified protocols:

Early Childhood (2–5 years):

Administered the Gesell Developmental Scale (GDS; Chinese normed edition, α=0.91), assessing gross/fine motor skills, adaptive functioning, language competence, and social-emotional development. Developmental Quotient (DQ) scores ≥85 indicate age-typical cognition per WHO guidelines.

School-Age (6–12 years):

Utilized the Wechsler Intelligence Scale for Children-Fourth Edition (WISC-IV; Chinese standardization, Yang et al., 2013, α=0.93), generating Full-Scale IQ scores. Cognitive impairment was operationalized using DSM-5 criteria: mild/typical: IQ/DQ ≥85 (-1 SD), significant deficit: IQ/DQ <85.

### Statistical analysis

Data analysis was performed between June and October 2021 using IBM SPSS Statistics 25.0 (Armonk, NY). Continuous variables were expressed as means ± standard deviations, and categorical variables as frequencies (%). Initial comparisons between ASD children with and without motor impairment employed Student’s t-tests for continuous measures and Pearson’s chi-square tests for categorical variables. Generalized linear models with a binomial distribution and logit link function were constructed to quantify associations between socioeconomic indicators (maternal education, household income) and motor dysfunction. Unadjusted models were followed by two progressive adjustment strategies: Model 1 controlled for demographic confounders (child’s age, sex, singleton status, maternal age); Model 2 additionally incorporated neurodevelopmental covariates (handedness, CARS total score). Sensitivity analyses addressed sex-specific effects through male-only subsample (n=142) reanalysis. Results were reported as adjusted odds ratios (aOR) with 95% confidence intervals derived from two-tailed tests, with statistical significance set at *p*<0.05. Comprehensive analytical outputs are detailed in **Supplementary Table 1**.

## Results

The analytical population comprised 165 children (3 excluded due to incomplete motor assessment data), with 53.3% (95% CI: 46.1–60.9%) meeting criteria for probable developmental coordination disorder. Participants’ mean age was 4.81 ± 1.55 years (range: 2–11), showing male predominance (83.0%). Family characteristics revealed 62.4% had siblings, 65.5% exhibited right-hand dominance, and 46.1% resided in households with a per capita monthly income below 5,000 CNY (≈790 USD; 2021 exchange rate). Cognitive functioning distribution indicated 67.9% scored ≥85 on standardized developmental/intelligence measures. Full demographic and clinical characteristics are detailed in **Table 1**.

**Table 1:** Demographic factors of the participants across gender.

Characteristics		Boys (n=137)	Girls (n=28)	*P* value
Age (year)		4.54 (0.16)	4.37 (0.35)	0.67
The only child (%)				
	Yes	54 (39.4)	8 (28.6)	0.28
	No	83 (60.6)	20 (71.4)	
Diagnosed age (year)		3.02 (0.10)	3.13 (0.21)	0.61
Right-handedness (%)				0.66
	Yes	91 (66.4)	9 (56.3)	
	No	46 (33.6)	17 (60.7)	
Maternal age (years)		34.27 (0.36)	31.57 (0.81)	0.003*
Maternal college degree or above (%)				0.84
	No	59 (43.1)	13 (46.4)	
	Yes	78 (56.9)	15 (53.6)	
Per capita monthly income of the family (%)				0.81
	<5000 yuan	62 (45.3)	14(50.0)	
	5000–8000 yuan	44 (32.1)	7 (25.0)	
	>8000 yuan	31 (22.6)	7 (25.0)	
Grandparenting				0.47
	Yes	54 (39.4)	9 (32.1)	
	No	83 (60.6)	19 (67.9)	
DCD positive (%)				0.98
	Yes	73 (53.3)	15 (53.6)	
	No	64 (46.7)	13 (46.4)	
The total score of CARS		35.64 (0.75)	34.07 (1.66)	0.39
Cognitive Level	Normal-mild deficit	54 (39.4)	12 (42.9)	0.209
	Moderate-very severe deficit	54 (39.4)	14 (50.0)	
	Unable to complete the test	29 (21.2)	2 (7.1)	

*p<0.05.

DCD, developmental coordination disorder.

Compared with girls, mothers of boys had an older maternal age at childbirth (34.27 ± 0.36 vs. 31.57 ± 0.81, boys, girls, respectively, *p* = 0.003). There is no gender difference in age, if the only child in the family, diagnosed age, right-handedness, maternal education level, per capita monthly income of the family, and probable DCD positive rate.

Compared with the ASD-only group, the ASD- probable DCD group had younger age (4.97 ± 0.20 vs. 4.07 ± 0.19 years, ASD-only vs. ASD- probable DCD, respectively, *p* = 0.007, **Table 2**), higher maternal college degree or above rates (46.8% vs.64.8%, ASD-only vs. ASD- probable DCD, respectively, *p* = 0.02, **Table 2**), more proportion of <5000 yuan per capita monthly family income (41.6% vs. 50.0%, ASD-only vs. ASD- probable DCD, respectively, *p*<0.001, **Table 2**), and the higher score of CARS (32.28 ± 0.95, 38.20 ± 0.88, ASD-only vs. ASD- probable DCD, respectively, *p*<0.001, **Table 2**). There is no group difference in gender, if the only child in the family, diagnosed age, right-handedness, maternal education level, and cognitive level (all *p*>0.05).

**Table 2:** Socio-demographic factors between the normal and probable DCD group.

Characteristics		ASD-only (n=77)	ASD- probable DCD (n=88)	*P* value
Gender (%)				0.97
	Boys	64 (83.1)	73 (83.0)	
	Girls	13 (16.9)	15 (17.0)	
Age (year)		4.97 (0.20)	4.07 (0.19)	0.007*
Only child (%)				0.32
	Yes	32 (41.6)	30 (34.1)	
	No	45 (58.4)	58 (65.9)	
Diagnosed age (year)		3.10 (0.12)	2.98 (0.12)	0.49
Right-handedness (%)				0.13
	Yes	55 (71.4)	53 (60.2)	
	No	22 (28.6)	35 (39.8)	
Maternal college degree or above (%)				0.020*
	No	41 (53.2)	31 (35.2)	
	Yes	36 (46.8)	57 (64.8)	
Per capita monthly income of the family (%)				0.008*
	<5000 yuan	32 (41.6)	44 (50.0)	
	5000–8000 yuan	19 (24.7)	32 (36.4)	
	>8000 yuan	26 (33.8)	12 (13.6)	
Grandparenting				0.40
	No	45 (58.4)	57 (64.8)	
	Yes	32 (41.6)	31 (35.2)	
The total score of CARS		32.28 (0.95)	38.20 (0.88)	<0.001*
Cognitive Level	Normal-mild deficit	36 (46.8)	30 (34.1)	0.099
	Moderate-very severe deficit	25 (32.5)	43 (48.9)	
	Unable to complete the test	16 (20.7)	15 (17.0)	

*p<0.05.

ASD, autism spectrum disorder; DCD, developmental coordination disorder; CARS, Childhood Autism Rating Scale.

The likelihood of ASD-probable DCD was higher among lower per capita monthly income of the family (5000–8000 yuan: Odds ratio [OR] 3.65, 95% confidence interval [CI] 1.50-8.88, <5000 yuan: OR 2.98, 95%CI 1.31-6.78, **Table 3**, crude model), compared with per capita monthly income of family >8000 yuan. After adjusting for age, gender, one child, maternal education level, and if only child, the 5000–8000 yuan group (OR 4.82, 95%CI 1.93-11.99, **Table 3**, adjusted model 1) and < 5000 yuan group (OR 4.12, 95%CI 1.73-9.81, **Table 3**, adjusted model 1) were still more likely classified as ASD- probable DCD. Additionally adjusted total score of CARS, odds of ASD- probable DCD were higher among children whose family had 5000–8000 yuan (OR 4.77, 95%CI 1.71-13.30, **Table 3**, adjusted model 2) and <5000 yuan per capita monthly income (OR 3.62, 95%CI 1.36-9.61, **Table 3**, adjusted model 2). The likelihood of ASD- probable DCD was higher when CARS score was higher (OR: 1.09, 95%CI:1.05-1.13, **Table 3**, crude model) and the results remain significant after adjusted child age, gender, one child, maternal education level, per capita family monthly income, if right-handedness, and if the only child (OR: 1.11, 95%CI:1.06-1.16, **Table 3**, adjusted model 2). Maternal without a college degree (OR: 0.33, 95%CI: 0.15-0.71, adjusted model 2, **Table 3**) showed a lower likelihood of having a child ASD- probable DCD report, compared with mothers who had a college degree or above. ASD children who had moderate to very severe cognitive deficits also had an increased likelihood to be classified as ASD- probable DCD (OR: 2.62, 95%CI 1.19-5.78, adjusted model 2, **Table 3**), compared with those who were cognitively normal or had mild deficits.

**Table 3:** Association between DCD positive and socio-demographic factors among ASD children.

		Crude model ^a^	Adjusted model 1 ^b^	Adjusted model 2 ^c^
Per capita monthly income of the family (%)				
	<5000 yuan	2.98 (1.31-6.78) *	4.12 (1.73-9.81) *	3.62 (1.36-9.61) *
	5000–8000 yuan	3.65 (1.50-8.88) *	4.82 (1.93-11.99) *	4.77 (1.71-13.30) *
	>8000 yuan	1	1	1
Maternal college degree or above (%)				
	Yes	1	1	1
	No	0.48 (0.26-0.89)*	0.43 (0.22-0.83)*	0.33 (0.15-0.71)*
Right-handedness (%)				
	Yes	1	1	1
	No	1.65 (0.86-3.17)	1.72 (0.85-3.47)	2.30 (0.99-5.33)
The total score of CARS		1.09 (1.05-1.13)*	1.10 (1.06-1.15) *	1.11 (1.06-1.16) *
Cognitive level (%)	Normal or mild deficit	1	1	1
	Moderate to very severe deficit	2.62 (1.24-5.56)*	2.62 (1.24-5.56)*	2.62 (1.19-5.78)*
	Unable to complete the test	2.09 (0.80-5.48)	2.10 (0.80-5.48)	2.20 (0.80-6.05)

*p<0.05.

^A^crude model had no adjustment.

^b^Adjusted child age, gender, maternal education level, and if the only child of the family.

^c^Adjusted variants in adjusted model 1 plus the total score of CARS, if right-handedness.

ASD, autism spectrum disorder; DCD, developmental coordination disorder; CARS, Childhood Autism Rating Scale.

## Discussion

This study investigated socioeconomic disparities in motor dysfunction among Chinese children with autism spectrum disorder (ASD), revealing two counterintuitive patterns: lower household income and higher maternal education both demonstrated independent associations with increased risk of motor impairment. Notably, the severity of core autism symptoms and presence of cognitive deficits emerged as stronger predictors of motor dysfunction compared to socioeconomic factors, suggesting neurobiological mechanisms may play predominant roles in motor development within this population. These findings challenge conventional socioeconomic-health paradigms in neurodevelopmental research, highlighting the necessity for integrated models that account for both environmental influences and endogenous biological pathways. To comprehensively interpret these complex findings, we situate our discussion within Bronfenbrenner’s Bioecological Theory of Human Development (11), which provides a cohesive framework for understanding the multilevel, interacting systems that shape the development of children with ASD.

The observed association between lower per capita family income and elevated motor coordination impairment can be interpreted through the lens of the bioecological model’s microsystem and exosystem​. As motor dysfunction demonstrates strong neurodevelopmental linkages with core autistic traits (31), this finding reflects broader patterns of social inequality affecting children with ASD. Existing evidence documents socioeconomic status (SES) gradients in autistic symptom severity (32–34) and behavioral challenges, compounded by systemic barriers to early diagnosis (35, 36) and limited access to developmental support services (1). These intersecting disadvantages create cumulative intervention delays that may exacerbate neuromotor impairments through multiple pathways: reduced exposure to motor-stimulating environments, diminished therapeutic resource allocation, and heightened psychosocial stressors impeding motor skill acquisition. ​Simultaneously, factors at the exosystem level, such as parental workplace pressures reducing engagement capacity or neighborhood characteristics limiting safe play spaces, can further constrain the child’s immediate environment and opportunities for motor development.

When interpreting the observed association between lower household socioeconomic status (SES) and greater motor dysfunction in children with ASD, the possibility of ​reverse causality​ must be prominently considered alongside the hypothesized causal pathway. It is plausible that the causal direction operates in reverse: more severe motor impairments could impose a substantial economic burden on families, thereby reducing SES (37). Key mechanisms may include ​reduced parental workforce participation​ due to caregiving demands, ​increased therapeutic expenditures, and costs of ​specialized equipment and home modifications​ (37). Our cross-sectional data cannot empirically determine the temporal ordering of these variables. Therefore, while the association is clear, we cannot conclude whether low SES contributes to motor deficits or if significant motor impairments lead to a decline in family SES through the pathways noted above. Future longitudinal studies tracking SES and motor skills over time are essential to disentangle this causal relationship.

Notably, while socioeconomic influences on motor development have been extensively documented in neurotypical populations (8, 10, 12, 13, 15–17, 20–22, 24, 38–41), this investigation constitutes the first empirical exploration of SES-motor relationships in autism spectrum disorder (ASD). Convergent evidence from typical development reveals robust associations between lower SES and compromised motor competence across domains: gross motor coordination (8, 10, 12–16, 18–20, 40, 41), fine motor precision (Comuk-Balci et al., 2016; Playford et al., 2017), and visuomotor integration (Ferreira et al., 2018; Özal et al., 2020). These patterns resonate with our ASD group findings, suggesting potential transdiagnostic mechanisms operating across ecological systems.

Emerging evidence delineates SES-mediated environmental mechanisms through which motor development trajectories become biologically embedded, ​a process central to the bioecological model’s concept of proximal processes​. The quality of home motor stimulation—operationalized by spatial organization for movement, availability of adaptive play materials, and caregiver-facilitated motor engagement (9) —exhibits graded associations with motor competency (42). Specifically, multidimensional home environmental adequacy (physical space parameters, developmentally calibrated toys, and intentional motor skill scaffolding) proves essential for normative motor maturation (10). Socioeconomic deprivation constrains access to motor-optimizing resources: safe outdoor play areas, proprioceptive-enriching equipment, and structured movement programs. This deprivation cascade interacts synergistically with ASD-related sensorimotor vulnerabilities (41), amplifying phenotypic expression of coordination deficits through gene-environment interplay mechanisms ​within the developing child’s immediate microsystem.

The economic repercussions of ASD diagnosis establish a bidirectional adversity between socioeconomic status and child development, demonstrating dynamic interplay across the chronosystem (timing of diagnosis relative to family resources) and exosystem (healthcare policies)​. Diagnostic confirmation precipitates substantial household financial strain through multiple channels: direct medical expenditures, specialized educational requirements, caregiver productivity losses, and long-term occupational adjustments (43). Empirical data reveal cross-national consistency in this economic impact - U.S. households experience a 14% income reduction post-diagnosis (44), while Chinese families incur annual losses averaging CNY 44,077 (≈USD 7,226 at 2015 exchange rates (45). Particularly in China’s evolving healthcare system, where 76% of ASD-related costs remain out-of-pocket (45), these fiscal pressures disproportionately constrain low-income families’ capacity to provide motor-enriching interventions. This creates a negative feedback loop within the bioecological model:​​ financial strain (exosystem) limits access to resources in the microsystem (movement therapies, adaptive equipment), potentially exacerbating motor deficits that may further increase caregiving demands and economic burdens, ​illustrating the theory’s principle of bidirectional influences​.

Paradoxically, our analyses revealed an inverse association between maternal education attainment and motor proficiency in children with ASD, a pattern contradicting established SES-motor relationships in neurotypical populations (15, 18, 40). This counterintuitive finding persisted in male-specific sensitivity analyses (**eTable II**), suggesting potential ASD-specific mechanisms. When interpreting this result, ​several non-mutually exclusive explanations warrant consideration, acknowledging the *post-hoc* and speculative nature of these interpretations in the absence of direct empirical data from our study​.

Within the bioecological framework, a culturally-mediated hypothesis​ also arises. The model’s macrosystem, which encompasses cultural norms, provides one lens for interpretation. It is conceivable that in Chinese sociocultural contexts, higher maternal education could be associated with increased workforce participation (affecting microsystem interactions) and a greater reliance on grandparental caregiving (62% vs. 34%; *p*<0.001). Some observational studies suggest that grandparental care may involve reduced motor-enriching play​ (22), potentially reflecting intergenerational differences in parenting practices. For children with ASD, whose motor development may benefit from targeted engagement, this caregiving pattern is one hypothesized pathway through which macrosystem factors might influence the microsystem (15). However, it is critical to emphasize that the “grandparenting hypothesis” remains ​a speculative interpretation​ pending direct investigation. The relative contribution of these potential mechanisms—biases versus culturally-mediated caregiving patterns—cannot be discerned from the present data and represents a crucial direction for future research.

Besides, we acknowledge that the observed association between higher maternal education and greater motor deficits could be influenced by diagnostic and reporting biases (46). More educated mothers may seek clinical evaluations more proactively, which is associated with the inclusion of children with milder motor impairments who might otherwise remain undiagnosed in less resource-equipped families (47). This could artifactually inflate the association between maternal education and motor dysfunction (47). On the other hand, ​reporting bias​ may arise if higher parental education levels lead to stricter developmental expectations, resulting in more critical ratings on motor assessments like the DCDQ, independent of actual child performance (48). While these biases do not invalidate the grandparenting hypothesis, they highlight the need for caution in causal interpretation. Future studies should combine objective motor assessments with caregiver reports and incorporate mediation analyses to disentangle these mechanisms.

Although we have theorized that lower SES may be associated with poorer motor outcomes through various bioecological pathways, ​we must seriously consider the possibility of reverse causality, due to the cross-sectional nature of our study design. Children with more severe motor deficits (potentially linked to greater core autistic symptoms or co-occurring intellectual disability) place higher demands on familial resources (47), linking to reduced parental workforce participation, increased therapeutic expenditures, and consequently, a decline in household socioeconomic status (SES) (49–52). ​This alternative pathway cannot be ruled out with our present data, where SES and motor function were assessed concurrently. Future longitudinal studies, measuring SES and motor skills at multiple time points from early development, are essential to disentangle the temporal ordering and clarify the causal mechanisms underlying this complex relationship.

Our findings substantiate the intrinsic neurodevelopmental linkages (the biological component of the bioecological model) between motor dysfunction and core autism pathology, with both symptom severity and cognitive impairment demonstrating robust associations with motor deficits (4). This triadic relationship aligns with the pervasive neural connectivity anomalies characterizing ASD (3), wherein early motor delays (2) may serve as prodromal markers preceding social-communication symptom emergence. Building upon the sensorimotor integration framework proposed by Bhat (4), our results corroborate motor impairment as a transdiagnostic manifestation of neuropathological processes underlying ASD heterogeneity. The child’s own biological characteristics are central to how they perceive and interact with their environment across all systems​.

These converging lines of evidence underscore the clinical imperative for dual-focused surveillance: 1) Implementing routine motor screening in toddlers exhibiting developmental red flags, regardless of social communication concerns; 2) Integrating motor proficiency assessments into standard ASD evaluation protocols. Early identification of motor vulnerabilities could enable preemptive intervention targeting cerebellar-striatal circuits through evidence-based motor rehabilitation paradigms, potentially modulating downstream social-cognitive trajectories in this population. ​From a bioecological perspective, such early interventions represent a targeted manipulation of the microsystem and mesosystem (e.g., therapy settings, home-program collaboration) during a critical period of the chronosystem, with the potential to alter the child’s developmental trajectory positively​.

## Strength

This study provides a novel perspective by introducing Bronfenbrenner’s bioecological model to understand the socioeconomic disparities in motor development among Chinese children with ASD. The findings indicate that lower household income (exosystem/macrosystem) significantly increases the risk of motor impairment, while higher maternal education may be associated with motor deficits through culturally mediated caregiving patterns. Crucially, these socioeconomic factors interact with the child’s own neurobiological characteristics, such as symptom severity and cognitive impairment, revealing a complex interplay between environmental and endogenous mechanisms. Clinically, these findings support the routine integration of standardized motor assessments into ASD surveillance systems for early identification, and emphasize the need for targeted motor rehabilitation programs for children from low SES backgrounds. Future interventions should be tailored to the Chinese context, optimizing intergenerational caregiving patterns and developing cost-effective community-based motor promotion programs that integrate micro-, meso-, and exosystems to comprehensively support child development.

## Limitation

A key limitation of our study is its cross-sectional design, which, as correctly noted, precludes causal inference and direct examination of underlying mechanisms. We did not measure potential mediators such as parenting practices or environmental enrichment, which compromised the mechanism exploration. Although variables such as parental marital status, urban/rural residence, household registration status (hukou) (Deng and Wang, 2023), and regional economic indicators (e.g., provincial GDP, cost-of-living indices) (Li and Ma et al., 2024) are recognized as critically influential in shaping autism-related outcomes and healthcare access disparities in China, these factors were not included in our analysis due to constraints in data availability and study design. For instance, ​hukou status​ fundamentally determines resource allocation in education and healthcare systems, potentially modulating rehabilitation service utilization (Zhu and Österle, 2017) ​.

Regional economic disparities​ could refine SES measurements by accounting for geographic variations in purchasing power and service density, while ​urban/rural residence​ often correlates with profound differences in healthcare infrastructure and community support networks (Zhu and Österle, 2017). ​ Although we have contextualized our household income categories against China’s per capita GDP, we recognize that our study did not fully adjust for intra-regional economic disparities across Southern China. Factors such as district-level variations in GDP per capita and cost-of-living differences could influence the precise socioeconomic positioning of families (53).

The grandparenting hypothesis—positing that higher maternal education may link to greater reliance on grandparental caregiving, potentially affecting motor stimulation—remains a *post-hoc* interpretation lacking direct empirical support in our study due to uncollected data on primary caregivers, grandparent involvement, or time allocation. This speculative pathway thus requires validation in future research that directly measures caregiving arrangements. Future studies would benefit from incorporating city- or district-level economic indices to create a cost-of-living-adjusted income metric for more precise SES measurement, and collect direct measures of caregiving patterns (e.g., grandparent involvement, time allocation) to test the grandparenting hypothesis empirically.

Parental marital status​ may further influence family dynamics and support structures affecting a child’s developmental environment (54). Their omission limits our ability to fully contextualize the observed SES-motor relationships within China’s unique sociocultural (30)l landscape. Future research should prioritize integrating these variables to enable a more nuanced, multi-level analysis of their direct and interactive effects on motor development in ASD, thereby enhancing the generalizability and policy relevance of findings.

We acknowledge that the rehabilitation center-based sampling strategy employed in our study may introduce ​selection bias (55), potentially leading to the ​underrepresentation of economically disadvantaged families​ who face barriers in accessing specialized services and ​children with milder motor impairments​ who are less likely to seek intensive rehabilitation (37). Consequently, our findings primarily reflect SES-motor associations ​among children with ASD already engaged in rehabilitation services, and the generalizability of these results to underserved populations (e.g., those in remote areas or with limited resources) may be limited. We hypothesize that the true magnitude of socioeconomic disparities in motor dysfunction could be ​even more pronounced in a population-based sample​ that encompasses the full spectrum of service access and socioeconomic diversity. Thus, future research should prioritize ​community-based sampling approaches​ to minimize selection bias and provide a more comprehensive understanding of these relationships.

## Conclusion

This study provides a ​novel bioecological perspective​ on socioeconomic disparities in motor development among Chinese children with ASD. We demonstrate that ​lower household income​ (Exosystem/Macrosystem) significantly increases motor impairment risk, while ​higher maternal education​ correlates with deficits through culturally-mediated caregiving patterns (Macrosystem/Chronosystem). Crucially, these socioeconomic factors ​interact with the child’s neurobiological profile​ (symptom severity, cognitive impairment), highlighting a ​complex interplay between environmental and endogenous mechanisms.

​Clinically, our findings advocate for routinely incorporating standardized motor assessments into ASD surveillance to enable early detection​. ​Targeted motor rehabilitation programs are particularly crucial for children from low-SES backgrounds to disrupt the cycle of socioeconomic disadvantage and neurodevelopmental exacerbation. Future interventions should address China-specific dynamics, including ​optimizing intergenerational caregiving patterns​ and ​developing cost-effective, community-based motor enrichment programs​ that actively integrate Micro-, Meso-, and Exosystems to holistically support child development. These findings ​challenge conventional SES-health paradigms​ and provide a ​theoretical basis for targeted policy interventions​ aimed at multilevel support systems for economically disadvantaged ASD families in China.

## Data Availability

The raw data supporting the conclusions of this article will be made available by the authors, without undue reservation.
