# Supplementary material for: Socioeconomic status and motor coordination function among children with autism
Source: Front Psychiatry. 2025 Nov 26;16:1619918. doi: 10.3389/fpsyt.2025.1619918 (PMC12689961; doi:10.3389/fpsyt.2025.1619918)
Supplement: Supplementary file 1 [file Table1.docx]

**Supplementary materials**

eTable Ⅰ. Previous Epidemiological Studies of Socio-economic Status and motor function of children.

| Author (year) | Study design | Sample size | Exposure | Age of sample | Study setting | Tools of motor assessment | Outcomes | Main findings |
| --- | --- | --- | --- | --- | --- | --- | --- | --- |
| Armstrong-Carter et al.(2021)^1^ | Longitudinal | 1,058 | Home stimulation | 4 years | Rural Pakistan | Unknown | Fine motor skills | Home stimulation at 18 months was positively associated with change in fine motor skills from ages 2 to 4, over and above family socioeconomic resources. |
| Gosselin, V et al. (2021)^2^ | Cross-sectional | 2,654 | School-level socioeconomic status | 6-12 years | Quebec, Canada | Product-oriented tests | Low motor competence | Children in high SES schools displayed the highest level of motor competence for agility, balance and coordination |
| Hairol, M et al. (2021)^3^ | Cross-sectional | 435 | Socio-economic factors | 5.08-6.83 years | Malaysia | the Beery-Bucktenicka Developmental Test | Visual-Motor Integration | Low-income families, low maternal education level, public preschools, started preschool at the elder age were more likely to obtain lower than average VMI scores. |
| Lopes, V. P et al. (2021)^4^ | Cross-sectional | 181 | Socio-economic status, living space, educational practices | 6.10±0.47 years | Portugal | the standing long jump, distance, throw of a tennis ball, and 20 m dash | Motor competence | Socio-economic variables, sibling characteristics/brotherhood relationship, type of housing/living space, child’s interaction with peers, and educational practices within the family, adequately explain the variance in motor competence in children aged five to six years |
| Möller,S et al. (2021)^5^ | Cross-sectional | 2,106 | Socio-economic status | 4-17 years | Leipzig, Germany | Pushups, standing long jump, backward balancing, jumping, side-to-side, forward bend | Motor skill | Higher socioeconomic status were significantly related to better motor skills. |
| Fink, G et al. (2020)^6^ | Multi-center, cross-sectional | 4,649 | Income contexts | 0-35 months | Cambodia, Chile, Ghana, Guatemala, Lebanon, Pakistan, the Philippines and the USA | CREDI | Age of motor milestone attainment, score of CREDI | Local socioeconomic context strongly predict average motor and language development of children under the age of 3years. |
| Kwon, S et al.(2020)^7^ | Cross-sectional | 329 | Socioeconomic status | 3-5 years | The USA | TGMD-2 | Gross motor skills | Children living below the poverty threshold were more likely to have a higher gross motor quotient |
| Özal, C et al.(2020)^8^ | Cross-sectional | 2,042 | Family and environment characteristics | 6 years above | Ankara, Turkey | the Denver II Developmental Test for Turkey | Gross motor developmental | Increasing maternal age and education were associated with later achievement in several items after age 12 months, while socioeconomic status did not show a clear effect. |
| Brian, A. et al. (2019)^9^ | Cross-sectional | 580,296 | Sex, race, geographic region, socioeconomic status | 3-6 years | The USA | TGMD-2 | Developmental delay in motor competence | No significant findings. |
| Villar, J. et al. (2019)^10^ | Multi-center, Cross-sectional | 1,307 | Diverse geographical locations | 2 years old | Brazil, India, Italy, Kenya and UK. | WHO motor milestones and visual tests | Age of motor milestones achievement | No significant findings. |
| Ferreira L. et al. (2018)^11^ | Cross-sectional | 707 | Home environment and socio-economic factors | 6-10 years | Southern Brazil | BOT-2, MP | Motor development | Motor development increased as SES increased, the best model used SES as the predictor, HOME as the mediator, and AGE as the moderator variable and explained 17% of the variance in motor development. |
| Veldman, S. L. C et al. (2018)^12^ | Cross-sectional | 335 | Socio-demographics factors | 11-29 months | Australian | PDMS-2 | Gross motor skills | Gross motor skills were negatively associated with age and a higher socio-economic status |
| Playford, C et al. (2017)^13^ | Longitudinal Study | 32,238 | Socioeconomic position | Preschool | Scotland, UK | A series of tasks | Fine motor, gross motor | Socioeconomic position was a strong predictor of development of hearing and language, vision and fine motor skills, and social skills but not gross motor skills. |
| Kakebeeke, T. H. et al. (2017)^14^ | Corhot study | 476 | Socioeconomic status | 30-80 months | Switzerland | Pegboard, repetitive hand and repetitive finger tasks | Motor speed, contralateral associated movements | The influence of physical activity and socioeconomic status on motor proficiency was small. |
| Oberer N. et al. (2017)^15^ | Cross-sectional | 156 | Socioeconomic status | 68-87 months | Switzerland | MABC-2 , jumping sideways, moving sideways, one-leg-stand | Motor skills | No significant finding regarding SES and motor skills. |
| Comuk-Balci, N et al. (2016)^16^ | Cross-sectional | 2,038 | Family factors | 0-82 months | Turkey | the Denver developmental screening test | Fine motor skills | Female gender, higher maternal age, especially in children older than 24 months, and higher maternal education were associated with earlier accomplishment of fine motor items. Higher socioeconomic status was correlated with fine motor skills more noticeably at young ages. |
| Klein, M et al. (2015)^17^ | Cross-sectional | 1,389 | Socio-economic status | 7-18 years | Germany | 20-m sprint, standing long jump and a 6-minute run | Motor performance | Students with a higher SES exhibit a higher motor performance compared with that of lower SES. |
| Morley, D. et al. (2015)^18^ | Cross-sectional | 369 | Socioeconomic status | 4-7 years | UK | BOT-2. (brief form) | The standardized scoring of the BOT-2 | High socioeconomic status significantly outperformed middle and/or low socioeconomic status for total, fine and gross motor proficiency. |
| Valentini N. C. et al. (2015)^19^ | Cross-sectional | 1,056 | Socioeconomic status | 4-10 years | Brazil | MABC-2 | The score of MABC-2 | Low SES enhances the risk of poor motor development of children. |
| Syrengelas D. et al. (2014)^20^ | Cross-sectional | 1,068 | Socioeconomic status | 7 days-19 months | Greece | AIMS | The score of AIMS | The educational level of the mother and also whether the infant was being raised by grandparents/babysitter were significantly associated with gross motor development. |
| Vandendriessche J. B. et al. (2012)^21^ | Cross-sectional | 1,955 | Socioeconomic status | 6-11 years | Belgium | Motor coordination test | Motor coordination | Tests of fitness and motor coordination showed a trend in favor of children from higher SES. |

[Abbreviation](javascript:;)

Caregiver Reported Early Development Instruments (CREDI), the Test of Gross Motor Development-Second Edition (TGMD-2), the Bruininks–Oseretsky Test of Motor Proficiency‐Second Edition (BOT-2), the Peabody Developmental Motor Scales Second Edition (PDMS-2), the Movement Assessment Battery for Children-2 (M-ABC-2) , The Alberta Infant Motor Scale (AIMS)

eTable Ⅱ. Sensitive analysis for the association between per capita monthly income of the family and probable DCD positive among autism children (only included boys, n=137).

|  |  | Adjusted model^a^ |
| --- | --- | --- |
| Per capita monthly income of the family (%) | |  |
|  | <5000 yuan | 5.78 (1.77-18.84)* |
|  | 5000-8000 yuan | 8.43 (2.39-29.77)* |
|  | >8000 yuan | 1 |
| Maternal college degree or above (%) | |  |
| Yes | | 1 |
| No | | 0.33 (0.14-0.79)* |
| A total score of CARS | | 1.11 (1.05-6.53)* |
| Right handedness | |  |
| Yes | | 1 |
| No | | 2.63 (1.06-5.75)* |
| Cognitive level | |  |
| Normal or mild deficit | | 1 |
| Moderate to very severe deficit | | 1.07 (0.38-3.04) |
| Unable to complete the test | | 0.99 (0.29-3.04) |

*p<0.05

^a^ Adjusted variants included age, gender, if only child, total score of CARS, if right-handedness and cognitive level.

The group with per capita family income between 5000-8000yuan had the highest rate of the only child (27.6%, 51.0% and 39.5%, <5000 yuan,5000-8000yuan and >8000 yuan, respectively, p=0.029, Table Ⅲ), while the group with per capita family income above 8000 yuan had the lowest score of CARS (36.56±1.09, 36.68±1.10 and 31.62±1.18, <5000 yuan, 5000-8000 yuan and >8000 yuan, respectively, p=0.008, eTable Ⅲ) and lowest rate of DCD positive (57.9%, 62.7% and 31.6% <5000 yuan, 5000-8000yuan and >8000 yuan, respectively, p=0.008, Table Ⅲ). There is no group difference in gender, age, diagnosed year, right-handedness, maternal education level, and cognitive level (all p>0.05).

The autistic children who had a mother with a college degree or above also had higher rates DCD (60.6% vs. 43.7%, p=0.03, eTable Ⅳ) and grandparenting (46.8% vs. 26.8%, p=0.009, Table Ⅳ). There were no group differences in sex, age, age at diagnosis, whether only child, age at intervention, per capita monthly income of family, total CARS score, right-handedness, or cognitive level (all p>0.05).

eTable Ⅲ. Socio-demographic factors among different income of the family.

|  | | Per capita monthly income of the family | | | P  value |
| --- | --- | --- | --- | --- | --- |
|  |  | <5000 yuan  (n=76) | 5000-8000 yuan  (n=51) | >8000 yuan (n=38) |  |
| Gender (%) |  |  |  |  | 0.81 |
|  | Boys | 62 (81.6) | 44(86.3) | 31 (81.6) |  |
|  | Girls | 14 (18.4) | 7 (13.7) | 7 (18.4) |  |
| Age (year) |  | 4.68(1.84) | 4.41 (1.74) | 4.34 (1.68) | 0.543 |
| An only child (%) |  |  |  |  | 0.029* |
|  | Yes | 21 (27.6) | 26 (51.0) | 15 (39.5) |  |
|  | No | 55 (72.4) | 26 (49.0) | 23 (60.5) |  |
| Diagnosed age (year) |  | 3.07 (1.11) | 3.07 (1.18) | 2.92 (0.97) | 0.765 |
| Right handedness (%) |  |  |  |  | 0.366 |
|  | Yes | 49 (64.5) | 37 (72.5) | 22 (57.9) |  |
|  | No | 27 (35.5) | 14 (27.5) | 16 (42.1) |  |
| Maternal college degree or above (%) |  |  |  |  | 0.503 |
|  | No | 37 (48.7) | 20 (39.2) | 15 (39.5) |  |
|  | Yes | 39 (51.3) | 31 (60.8) | 23 (60.5) |  |
| Grandparenting |  |  |  |  | 0.28 |
|  | No | 50 (66.7) | 27 (52.9) | 25 (64.1) |  |
|  | Yes | 25 (33.3) | 24 (47.1) | 14 (35.9) |  |
| The total score of CARS |  | 36.56 (1.09) | 36.68 (1.10) | 31.62 (1.18) | 0.008* |
| Cognitive Level (%) | Normal-mild deficit | 25 (32.9) | 18 (35.3) | 23 (60.5) |  |
|  | Moderate-very severe deficit | 34 (44.7) | 23 (45.1) | 11 (28.9) |  |
|  | Unable to complete the test | 17 (22.4) | 10 (19.6) | 4 (10.5) |  |
| probable DCD positive (%) |  |  |  |  | 0.008* |
|  | Yes | 44 (57.9) | 32 (62.7) | 12 (31.6) |  |
|  | No | 32 (42.1) | 19 (37.3) | 26 (68.4) |  |

*P<0.05

DCD, developmental coordination disorder, CARS, Childhood Autism Rating Scale

eTable Ⅳ. Sociodemographic factors among different maternal education level.

|  | | Maternal college degree or above | | P  value |
| --- | --- | --- | --- | --- |
|  |  | No  (n=71) | Yes  (n=94) |  |
| Sex (%) |  |  |  | 0.690 |
|  | Boys | 58 (81.7) | 79 (84.0) |  |
|  | Girls | 13 (18.3) | 15 (16.0) |  |
| Age (year) |  | 4.42 (0.23) | 4.22 (0.20) | 0.507 |
| An only child (%) |  |  |  | 0.065 |
|  | Yes | 21 (29.6) | 41 (43.6) |  |
|  | No | 50 (70.4) | 53 (56.4) |  |
| Diagnosed age (year) |  | 3.05 (0.14) | 2.97 (0.12) | 0.682 |
| Intervention age (year) |  | 3.32 (0.15) | 2.94 (0.13) | 0.059 |
| Right handedness (%) |  |  |  | 0.195 |
|  | Yes | 43 (60.6) | 66 (70.2) |  |
|  | No | 28 (39.4) | 28 (29.8) |  |
| Per capita monthly income of the family (%) |  |  |  | 0.499 |
|  | <5000 yuan | 36 (50.7) | 39 (41.5) |  |
|  | 5000-8000 yuan | 20 (28.2) | 31 (33.0) |  |
|  | >8000 yuan | 15 (21.1) | 24 (25.5) |  |
|  |  |  |  |  |
| Grandparenting | Yes | 19 (26.8) | 44 (46.8) | 0.009* |
|  | No | 52 (73.2) | 50 (53.2) |  |
| The total score of CARS |  | 35.06 (1.08) | 34.93 (0.93) | 0.929 |
| Cognitive Level (%) |  |  |  | 0.524 |
|  | Normal-mild deficit | 26 (36.6) | 40 (42.6) |  |
|  | Moderate-very severe deficit | 29 (40.8) | 39 (41.5) |  |
|  | Unable to complete the test | 16 (22.5) | 15 (16.0) |  |
| probable DCD positive (%) |  |  |  | 0.030* |
|  | Yes | 31 (43.7) | 57 (60.6) |  |
|  | No | 40 (56.3) | 37 (39.4) |  |

*P<0.05

DCD, developmental coordination disorder, CARS, Childhood Autism Rating Scale

**eReferences:**

1.Armstrong-Carter E, Sulik MJ, Siyal S, Yousafzai AK, Obradović J. Early and concurrent home stimulation: Unique and indirect links with fine motor skills among 4-year-old children in rural Pakistan. *Dev Psychol* 2021;57(6):888-899.doi: 10.1037/dev0001185.PubMed: 34424007

2.Gosselin V, Leone M, Laberge S. Socioeconomic and gender-based disparities in the motor competence of school-age children. *J Sport Sci* 2021;39(3):341-350.doi: 10.1080/02640414.2020.1822585.PubMed: 32967566

3.Hairol MI, Nordin N, P Ng J, et al. Association between reduced visual-motor integration performance and socioeconomic factors among preschool children in Malaysia: A cross-sectional study. *Plos One* 2021;16(3):e0246846.doi: 10.1371/journal.pone.0246846

4.Lopes VP, Monteiro D. Socio-Cultural and Somatic Factors Associated with Children’s Motor Competence. *Journal of Functional Morphology and Kinesiology* 2021;6(2):54.doi: 10.3390/jfmk6020054

5.Möller S, Poulain T, Körner A, et al. Motor skills in relation to body-mass index, physical activity, TV-watching, and socioeconomic status in German four-to-17-year-old children. *Plos One* 2021;16(5):e0251738.doi: 10.1371/journal.pone.0251738

6.Fink G, McCoy DC, Yousafzai A. Contextual and socioeconomic variation in early motor and language development. *Arch Dis Child* 2020;105(5):421-427.doi: 10.1136/archdischild-2019-317849

7.Kwon S, O Neill M. Socioeconomic and Familial Factors Associated with Gross Motor Skills among US Children Aged 3–5 Years: The 2012 NHANES National Youth Fitness Survey. *Int J Env Res Pub He* 2020;17(12):4491.doi: 10.3390/ijerph17124491

8.Özal C, Bayoğlu B, Karahan S, Günel MK, Anlar B. Gross motor development of preschool children: effects of socioeconomic status and maternal education. *The Turkish Journal of Pediatrics* 2020;62(1):10.doi: 10.24953/turkjped.2020.01.002.PubMed: 32253861

9.Brian A, Pennell A, Taunton S, et al. Motor Competence Levels and Developmental Delay in Early Childhood: A Multicenter Cross-Sectional Study Conducted in the USA. *Sports Med* 2019;49(10):1609-1618.doi: 10.1007/s40279-019-01150-5

10.Villar J, Fernandes M, Purwar M, et al. Neurodevelopmental milestones and associated behaviours are similar among healthy children across diverse geographical locations. *Nat Commun* 2019;10(1).doi: 10.1038/s41467-018-07983-4

11.Ferreira L, Godinez I, Gabbard C, Vieira JLL, Caçola P. Motor development in school-age children is associated with the home environment including socioeconomic status. *Child: Care, Health and Development* 2018;44(6):801-806.doi: 10.1111/cch.12606

12.Veldman SLC, Jones RA, Santos R, Sousa-Sá E, Okely AD. Gross motor skills in toddlers: Prevalence and socio-demographic differences. *J Sci Med Sport* 2018;21(12):1226-1231.doi: 10.1016/j.jsams.2018.05.001

13.Playford CJ, Dibben C, Williamson L. Socioeconomic disadvantage, fetal environment and child development: linked Scottish administrative records based study. *Int J Equity Health* 2017;16(1).doi: 10.1186/s12939-017-0698-4

14.Kakebeeke TH, Zysset AE, Messerli-Bürgy N, et al. Impact of age, sex, socioeconomic status, and physical activity on associated movements and motor speed in preschool children. *J Clin Exp Neuropsyc* 2017;40(1):95-106.doi: 10.1080/13803395.2017.1321107.PubMed: 28548032

15.Oberer N, Gashaj V, Roebers CM. Motor skills in kindergarten: Internal structure, cognitive correlates and relationships to background variables. *Hum Movement Sci* 2017;52:170-180.doi: 10.1016/j.humov.2017.02.002

16.Comuk-Balci N, Bayoglu B, Tekindal A, Kerem-Gunel M, Anlar B. Screening preschool children for fine motor skills: environmental influence. *J Phys Ther Sci* 2016;28(3):1026-31.doi: 10.1589/jpts.28.1026.PubMed: 27134406

17.Klein M, Fröhlich M, Pieter A, Emrich E. Socio-economic status and motor performance of children and adolescents. *Eur J Sport Sci* 2015;16(2):229-236.doi: 10.1080/17461391.2014.1001876

18.Morley D, Till K, Ogilvie P, Turner G. Influences of gender and socioeconomic status on the motor proficiency of children in the UK. *Hum Movement Sci* 2015;44:150-156.doi: 10.1016/j.humov.2015.08.022

19.Valentini NC, Clark JE, Whitall J. Developmental co-ordination disorder in socially disadvantaged Brazilian children. *Child: Care, Health and Development* 2015;41(6):970-979.doi: 10.1111/cch.12219

20.Syrengelas D, Kalampoki V, Kleisiouni P, Konstantinou D, Siahanidou T. Gross motor development in full-term Greek infants assessed by the Alberta Infant Motor Scale: Reference values and socioeconomic impact. *Early Hum Dev* 2014;90(7):353-357.doi: 10.1016/j.earlhumdev.2014.04.011

21.Vandendriessche JB, Vandorpe BFR, Vaeyens R, et al. Variation in Sport Participation, Fitness and Motor Coordination With Socioeconomic Status Among Flemish Children. *Pediatr Exerc Sci* 2012;24(1):113-128.doi: 10.1123/pes.24.1.113.PubMed: 22433257
